# Relationship between family functioning and self-transcendence in patients with breast cancer: A network analysis

**DOI:** 10.3389/fpubh.2022.1028860

**Published:** 2022-11-17

**Authors:** Chunyan He, Tianqi Yang, Yang He, Sijin Guo, Yawei Lin, Chao Wu, Li Gao, Xufeng Liu, Shengjun Wu, Baohua Cao

**Affiliations:** ^1^Department of Nursing, Fourth Military Medical University, Xi'an, China; ^2^Department of Psychology, Fourth Military Medical University, Xi'an, China; ^3^Xijing Hospital Affiliated With Air Force Medical University, Xi'an, China

**Keywords:** breast cancer, self-transcendence, family functioning, network analysis, nursing

## Abstract

**Background:**

For patients with breast cancer, family functioning is an important factor affecting self-transcendence, which is a key source of happiness. However, network analysis studies of family functioning and self-transcendence are lacking, particularly among patients with breast cancer.

**Purpose:**

The present study investigated the network structure of family functioning and self-transcendence in patients with breast cancer and aimed to identify bridge items to provide some theoretical support for the improvement and intervention of self-transcendence in patients with breast cancer.

**Methods:**

A total of 294 patients with breast cancer were enrolled in our study. Self-transcendence was evaluated with the Self-Transcendence Scale. Family functioning was evaluated with the Family Adaptation, Participation, Growth, Affection, Resolution (APGAR) Scale. Network analyses were used for the statistical analysis.

**Results:**

In the network of family functioning and self-transcendence in patients with breast cancer, there were 22 edges across communities, of which the 5 strongest edges connected to the 5 dimensions of family functioning are “Adaptation” with “Enjoyment of hobbies”, “Participation” with “Life enjoyment”, “Growth” with “Acceptance of bodily changes”, “Affection” with “Life enjoyment”, “Resolution” with “Help acceptance”. “Adaptation” had the highest bridge expected influence value (0.30) in the family functioning community, while “Life enjoyment” had the highest bridge expected influence value (0.27) in the self-transcendence community.

**Conclusion:**

Complex patterns of associations existed in the fine-grained relationship between family functioning and self-transcendence in patients with breast cancer. From the perspective of network analysis, the “Adaptation” aspect of family functioning and the “Life enjoyment” aspect of self-transcendence may be the best targets for improving self-transcendence. These results have important implications to clinical practice, which provided potential targets for interventions to improve self-transcendence from the perspective of family functioning.

## Introduction

Breast cancer is one of the most common malignant tumors in women. In recent years, the incidence and death rates of breast cancer patients have been increasing year by year, and the age at diagnosis has gradually been decreasing (1). According to the International Agency for Research on Cancer (IARC) global cancer statistics report in 2020 (2), the incidence rate has surpassed that of lung cancer, with breast cancer becoming the “number one new cancer worldwide”. The theory of self-transcendence refers to the individual's ability to transcend the status quo by one's adjusting personal goals, perceptions, and behavioral activities; accepting oneself positively; establishing good interpersonal relationships; finding meaning in life; and expanding one's capabilities without denying oneself and one's existing values (3–5). Numerous studies have shown that self-transcendence is closely related to the spiritual health and quality of survival of cancer patients (6–9). The reason for this may be that patients with lower levels of self-transcendence are less capable in many aspects, that their physiological and psychological states are vulnerable to negative effects, and that it is difficult for them to adapt to the physical and mental stress caused by disease-related factors, leading them to be unable to actively cope with disease recovery and to have less confidence in treatment (10); on the other hand, patients with higher levels of self-transcendence can openly accept the fact that they have cancer, can accept and adapt to their physical and mental changes, and can adapt to the changes in their surroundings and adjust to the physical, emotional and spiritual stresses they face (11, 12). It is suggested that self-transcendence is a key element for cancer patients to integrate resources and solve problems through their own wisdom (5, 13), which is especially important for breast cancer patients (14, 15) because both the diagnosis and treatment process of breast cancer (e.g., surgery, radiotherapy) can cause different degrees of physical and psychological damage to patients. For example, from the initial cancer diagnosis to the postoperative changes in body image and the various adverse effects caused by radiotherapy and endocrine therapy, patients develop various negative emotions and often hold a series of pessimistic attitudes toward treatment and life (16). This leads to a plethora of psychological problems, such as low self-esteem, depression, anxiety and social avoidance, which in turn affects patients' level of self-transcendence (17–19). In recent years, numerous studies (17, 20, 21) have shown that improving patients' self-transcendence level is of great significance for improving patients' physical and mental health and quality of life. Therefore, in theory and in practice, it is important to determine how to improve the self-transcendence of patients with breast cancer.

Self-transcendence encompasses psychological, social, and spiritual aspects, and it engenders a sense of wellbeing in vulnerable individuals as their lives are coming to an end; it emphasizes the use of greater strength to find relief in difficult situations and a renewed awareness of negative events (22, 23). In studies of self-transcendence tests on different populations, Chinese and foreign scholars have found that individuals' self-transcendence is related to their age, gender, culture, beliefs, life experiences, personal character traits, health and disease treatment. The main correlates of self-transcendence are happiness, life orientation, self-esteem, hope, life goals, conformity, quality of life, and family caring, especially crucially, family caring (24, 25). Family caring is an important indicator to measure the status of family functioning and to evaluate the quality of life of patients, and it can reflect the subjective satisfaction of family members with family functioning (26), which is used to detect family functioning and is an important indicator of the operational effectiveness of family systems. Related studies have concluded that family functioning not only provides certain environmental conditions for the healthy development of family members' physiological, psychological, and social aspects, but also predicts individuals' psychological health level and subjective wellbeing level as well as self-transcendence level (19, 24). Researchers Holland and Mastrovito stated that the adaptation of breast cancer patients to breast cancer depends mainly on two factors, namely, the psychosocial environment and patients' health status, and previous studies have shown that family functioning plays a very important role in the psychological health of breast cancer patients (27). Family functioning refers to the ability of family members to maintain close relationships with each other, perform well in their family roles, cope with various family problems, adapt to new family practices, and communicate effectively with each other (24). Studies have found that family functioning can have a tremendous impact on the health of individuals, especially cancer patients, due to its ability to provide strong psychological and material support during times when individuals' health is at risk (25). Mainly caused by the differences between Chinese and Western cultures, traditional family beliefs in Chinese society lead breast cancer patients to rely mainly on family support and care during treatment; therefore, family functioning directly affects the quality of support for breast cancer patients, which is the most important component of social support (28, 29). In recent years, a large number of studies on breast cancer patients have shown a positive correlation between family functioning and self-transcendence; i.e., the better the family functioning is, the higher the patient's level of self-transcendence (19, 24, 30).

However, previous studies investigating the relationship between family functional characteristics and the level of self-transcendence in breast cancer patients through scale measurements have tended to consider self-transcendence as a whole when, in fact, self-transcendence includes psychological, social and spiritual aspects. In this way, the determination of correlation based on the total score disguises the relationship between different dimensions of variables. Second, the traditional statistical model cannot evaluate the relative importance of different interrelated nodes in the network by calculating indicators. Finally, in the variable research on family function and self-transcendence, spurious correlations among variables easily appear when there are more variables. For example, when two variables are correlated with other variables, even if the two variables are unrelated, the correlation may be statistically significant without controlling for other variables (31). Network analysis can reduce these to some extent by fitting the data using a Gaussian image model (i.e., biased correlation network) (32). More importantly, we applied the network analysis method for the first time to study the fine-grained relationship between family functioning and self-transcendence in breast cancer patients, expecting to find the best intervention targets and provide timely and targeted interventions to help breast cancer patients improve their self-transcendence by taking advantage of the network analysis method.

Network analysis is a promising statistical method based on mathematical analysis and visual representation of the interaction between complex variables. First, network analysis is data-driven and does not rely on prior assumptions about the relationship between variables (33), so it can be used to analyze the relationship between different variables in the mental process in a fine-grained manner. Second, network analysis allows the visualization of association patterns of different variables (34) and provides centrality indices to assess the relative importance of a given variable in a network (35). Among them, the term “community” is used to represent a set of psychological variables based on theory, and bridge centrality indices helps accurately capture the variables that play a key role in connecting communities (36). Thus, network analysis can provide indicators of bridge centrality to quantitatively compare the overall strength of connectivity among variables from a network system perspective, allowing for the exploration of correspondence between different entries of multiple variables. Compared with traditional correlation analysis methods, network analysis can using a regularization process to control the occurrence of spurious correlations (32).

In summary, this study was conducted based on a network analysis approach to analyze the relationship between family functioning and self-transcendence, and improvement in the level of self-transcendence in breast cancer patients. We constructed the network model and estimated the bridge centrality to detect the important role of some specific aspects of family functioning in improving self-transcendence in breast cancer patients and identified the variables connecting these two communities to provide targets for interventions to achieve self-transcendence in breast cancer patients.

## Materials and methods

### Participants

A total of 294 patients with breast cancer from three tertiary grade-A hospitals in Xi'an, China, participated in a cross-sectional descriptive survey between September 2021 and December 2021; the patients were recruited *via* the convenience sampling method (general information shown in Table 1). Patients who met the following inclusion criteria were included in the research: (a) had received their first confirmed pathologic breast cancer diagnosis; (b) were female; (c) were aged 18–60; (d) were conscious without cognitive dysfunction or communication disorder; and (e) gave informed consent and volunteered to participate in the research.

**Table 1:** Demographic characteristics of the patients with breast cancer (*n* = 294).

**Variables**	**Number (*n*)**	**Percentage (%)**
**Age (years)**
20–30	7	2.38
31–40	69	23.47
41–50	105	35.71
51–60	113	38.44
**Educational level**
Junior secondary and below	119	40.48
High school/junior college	119	40.48
Bachelor and above	56	19.04
**Occupation**
Enterprises/institutions	71	24.15
Laborer	56	19.05
Retired	81	27.55
Unemployed	86	29.25
**Marital status**
Married	272	92.52
Unmarried/divorced/widowed	22	7.48
**Family monthly income per capita (RMB)**
< 3,000	131	44.56
3,000–5,000	82	27.89
>5,000	81	27.55
**Place of residence**
Urban area	204	69.39
Rural area	90	30.61
**Live alone**
Yes	14	4.76
No	280	95.24
**Treatment stage**
Surgery	44	14.97
Chemotherapy	150	51.02
Radiotherapy	39	13.27
Other	61	20.74
**Surgical method**
Breast-conserving	72	24.49
Radical mastectomy	167	56.80
Breast reconstruction	55	18.71

The exclusion criteria were as follows: (a) recurrence or metastasis of breast cancer during treatment; (b) other malignancies and significant organ dysfunction; and (c) a previous history or family history of severe mental illness.

A rough estimation method of sample size was used: 5–10 times the number of study variables was used to calculate the sample size (37). There were 29 variables in the study; hence, the sample size of this study was 145–290 patients. Considering a loss rate of 20%, the minimum sample size of the study was 174 patients.

### Data collection

The researchers explained the purpose, significance, and content of the research and selected participants strictly according to the selection criteria; the participants anonymously completed a paper questionnaire. The paper-based questionnaire used a standard guideline and ensured confidentiality of all research data. After the questionnaire was collected, invalid questionnaires were screened. The elimination criteria were as follows: (a) incomplete questionnaire responses or (b) apparent regularity in the answers to the questionnaire or all identical answers. A total of 300 questionnaires were collected. Finally, 294 questionnaires were received, for an effective response rate of 98.00%. The research followed the ethical principles of the Declaration of Helsinki, and ethical approval (KY20192117-F-1) was obtained by the Ethics Committee of the First Affiliated Hospital of the Fourth Military Medical University.

### Measurements

#### The family APGAR scale

The Family APGAR Scale, developed by Dr. Smilkstein (38) and translated into Chinese by Lv and Gu (39), has 5 items. Each item represents one dimension of family functioning, and the 5 dimensions are: adaptation, participation, growth, affection, and resolution. All items are rated by on a 3-point Likert scale, where “rarely,” “sometimes,” and “often” are scored from 0 to 2. The total possible score is 10. Higher scores indicate better family functioning. A total score of 0–3 indicates severe family dysfunction, 4–6 indicates moderate family dysfunction and 7–10 indicates good family functioning. The Cronbach's α coefficient of the scale was 0.809, equating to good reliability.

#### The Chinese version of the self-transcendence scale

The C-STS, compiled by Reed (3) and revised by Zhang et al. (40), includes 15 items. In this study, each item is represented by its abbreviation for ease of description. Such as “Enjoyment of hobbies” represents “In my present life, I enjoy my existing hobbies or interests”. Each item is rated on a 4-point Likert scale (1 = “describes me not at all” to 4 = “describes me very much”), for a total score ranging from 15 to 60 points. The higher the score is, the higher the level of self-transcendence. A total score > 45 indicates a higher level of self-transcendence. The Cronbach's α coefficient of the scale was 0.881, indicating good reliability.

### Data analysis

SPSS 25.0 software was used to analyze sociodemographic characteristics, family functioning, self-transcendence and other descriptive data, and R 4.1.1 software was used to construct the network model and estimate the bridge centrality to identify the bridge items between family functioning and self-transcendence.

### Network model

The network of family functioning and self-transcendence was constructed by the R package qgraph (41). Least absolute shrinkage and selection operator (LASSO) (42) regularization and the extended Bayesian information criterion (EBIC) (43) were used to shrink minor edges to zero weight (44). Spearman's correlation was adopted, and the EBIC hyperparameter was set to 0.5. In the network, nodes represent different contents of family functioning and self-transcendence, lines represent statistical relationships between these contents, the influence on the correlation of any two nodes caused by other nodes was eliminated by statistical control (32). According to the subordination of items represented by the nodes, the nodes were grouped into different communities. The family functioning community consisted of adaptation, participation, growth, affection and resolution, while the self-transcendence community consisted of enjoyment of hobbies, acceptance of aging, involvement with people, adjustment to life, acceptance of bodily changes, experience sharing, meaning of the past, helping others, interest in learning, problem solving, death acceptance, meaning in faith, help acceptance, life enjoyment and letting go of the past. The accuracy test and the difference significance test of edge weight indices were conducted by the R package bootnet (44).

### Bridge centrality

The bridge centrality was estimated by R package network tools (36). The bridge expected influence (BEI) was estimated in the present study; the BEI of a node is defined as the sum of the connections between the node and all nodes from another community. The stability test and difference significance test of the BEI indices were conducted by the R package bootnet (44), and the stability was quantitatively evaluated by the correlation stability coefficient (CS-coefficient), whose acceptable value is larger than 0.25 (44).

## Results

### Descriptive statistics

Among the 294 patients, the mean (SD) age was 46.90 ± 8.72 years. In total, 40.48% had a junior high school education or below, 29.25% were unemployed, 92.52% were married, 44.56% had a monthly income < 3,000 yuan, 30.61% lived in rural areas, 4.76% lived alone, 51.02% were undergoing chemotherapy, and 56.80% had undergone radical mastectomy. The other characteristics of the participants are shown in Table 1. Moreover, the mean scores, standard deviations and abbreviations for each item for family functioning and self-transcendence are presented in Table 2.

**Table 2:** Mean scores, standard deviations and abbreviations for each item on self-transcendence and family functioning.

**Items**	**Abbreviation/dimensions**	**M**	**SD**	**BEI**
**Self-transcendence (ST)**		45.88	7.29	
T1: In my present life, I enjoy my existing hobbies or interests	Enjoyment of hobbies	2.94	0.86	0.13
T2: As I grow older, I can still accept myself	Acceptance of aging	3.34	0.72	0.00
T3: I still get involved with people or communities	Involvement with people	3.01	0.89	0.04
T4: I can make a good adjustment to my present life	Adjustment to life	3.19	0.77	0.01
T5: I can accept and adjust to changes in my bodily functions	Acceptance of bodily changes	3.04	0.73	0.13
T6: I am willing to share my wisdom or experience with others	Experience sharing	3.37	0.71	0.02
T7: I can find meaning in my past experience	Meaning of the past	3.15	0.72	0.05
T8: I will help others in some ways	Helping others	3.33	0.68	0.00
T9: My interest in learning never stops	Interest in learning	2.71	0.96	0.00
T10: When faced with something important, I can think beyond	Problem solving	2.91	0.73	0.00
the problem and solve it				
T11: I can accept that death is a part of life	Death acceptance	2.86	0.80	0.11
T12: I can find meaning in my faith	Meaning in faith	3.01	0.71	0.03
T13: When I am in need, I am willing to accept help from others	Help acceptance	3.08	0.79	0.12
T14: I enjoy the pace of my life right now	Life enjoyment	2.78	0.90	0.27
T15: I can let go of the gains and losses of the past	Letting go of the past	3.17	0.73	0.00
**Family functioning (FF)**		7.27	2.41	
F1: Sharing resources and satisfaction with the attention received	Adaptation	1.56	0.63	0.30
F2: Joint decision making and family communication when problem solving	Participation	1.43	0.65	0.15
F3: Emotional growth due to the freedom to change roles within the family	Growth	1.49	0.61	0.19
F4: Individual's satisfaction regarding family interactions and intimacy among family members	Affection	1.23	0.72	0.20
F5: Sharing time and satisfaction with the commitments established	Resolution	1.56	0.60	0.07

### Network estimation

**Figure 1:**
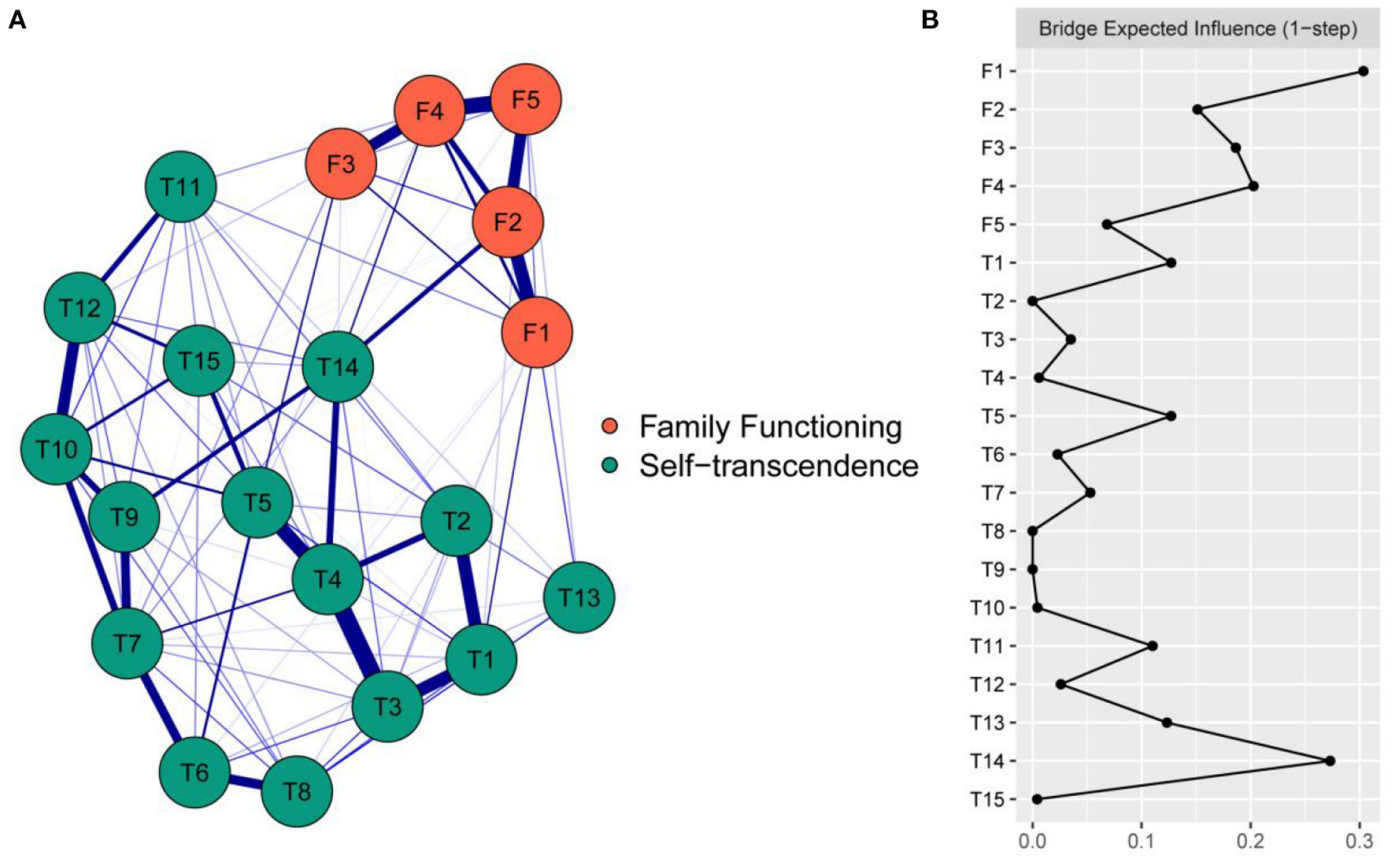
The network model of family functioning and self-transcendence and the bridge expected influence. **(A)** The network model of family functioning and self-transcendence. The nodes represent items of family functioning and self-transcendence, the blue edges represent connections between items, and thick lines and saturated colors represent close connections. In cross-community edges, the strongest correlation of F1 was with T1, the strongest correlation of F2 was with T14, the strongest correlation of F3 was with T5, the strongest correlation of F4 was with T14, the strongest correlation of F5 was with T13. **(B)** The bridge expected influence indices in the network of family functioning and self-transcendence (raw score). F1 had the highest bridge expected influence value (0.30) in the family functioning community, while T14 had the highest bridge expected influence value (0.27) in the self-transcendence community. F1 = adaptation; F2 = participation; F3 = growth; F4 = affection; F5 = resolution; T1 = enjoyment of hobbies; T2 = acceptance of aging; T3 = involvement with people; T4 = adjustment to life; T5 = acceptance of bodily changes; T6 = experience sharing; T7 = meaning of the past; T8 = helping others; T9 = interest in learning; T10 = problem solving; T11 = death acceptance; T12 = meaning in faith; T13 = help acceptance; T14 = life enjoyment; T15 = letting go of the past.

The network model is shown in Figure 1A. There were 22 edges (range from <0.01 to 0.14) across the family functioning and self-transcendence communities in the network. In the edges across communities, F1 “Adaptation” was positively correlated with 6 nodes of the self-transcendence community, namely, T1 “Enjoyment of hobbies”, T3 “Involvement with people”, T4 “Adjustment to life”, T6 “Experience Sharing”, T11 “Death acceptance” and T13 “Help acceptance”, among which the correlation with T1 “Enjoyment of hobbies” was the strongest (edge weight = 0.10). F2 “Participation” was positively correlated with 3 nodes of the self-transcendence community, namely, T10 “Problem solving”, T14 “Life enjoyment” and T15 “Letting go of the past”, among which the correlation with T14 “Life enjoyment” was the strongest (edge weight = 0.14). F3 “Growth” was positively correlated with 5 nodes of the self-transcendence community, namely, T3 “Involvement with people”, T5 “Acceptance of bodily changes”, T6 “Experience sharing”, T7 “Meaning of the past” and T14 “Life enjoyment”, among which the correlation with T5 “Acceptance of bodily changes” was the strongest (edge weight = 0.10). F4 “Affection” was positively correlated with 4 nodes of the self-transcendence community, namely, T5 “Acceptance of bodily changes”, T11 “Death acceptance”, T12 “Meaning in faith” and T14 “Life enjoyment”, among which the correlation with T14 “Life enjoyment” was the strongest (edge weight = 0.10); F5 “Resolution” was positively correlated with 4 nodes of the self-transcendence community, namely, T1 “Enjoyment of hobbies”, T3 “Involvement with people”, T13 “Help acceptance” and T14 “Life enjoyment”, among which the correlation with T13 “Help acceptance” was the strongest (edge weight = 0.03). See Supplementary Table 1 for more detailed information on the correlations among nodes in the network. For edges in the family functioning community, there were 10 edges ranging from 0.05 to 0.29, and the strongest correlation was between F4 “Affection” and F5 “Resolution”. For edges in the self-transcendent community, there were 66 edges ranging from <0.01 to 0.33, and the strongest correlation was between T4 “Adjustment to life” and T3 “Involvement with people”.

In the network of family functioning and self-transcendence, the 95% confidence interval of edge weights was narrow, which indicated that the accuracy of edge weights was acceptable (see Supplementary Figure 1). As the result of the difference significance test of the edge weight showed, the edge weight between F2 “Participation” and T14 “Life enjoyment” was the largest among the cross-community edges and was statistically larger than F5-T3 and F2-T10 (*P* < 0.05, see Supplementary Figure 2).

### Bridge expected influence

As shown in Figure 1B, in the family functioning community, F1 “Adaptation” had the largest BEI value (0.30), while in the self-transcendence community, T14 “Life enjoyment” had the largest BEI value (0.27). As shown in Supplementary Figure 3, with the reduction of the subsample, the average correlation of the BEI indices of the original sample and the subsample decreased, while the CS coefficient was 0.361, which was larger than 0.25 and indicated acceptable stability. As shown in Supplementary Figure 4, the BEIs of F1 “Adaptation” and T14 “Life enjoyment” were statistically larger than those of at least half of the other nodes (*P* < 0.05).

## Discussion

In this study, a network structure of the relationship between family functioning and self-transcendence in breast cancer patients was constructed by means of network analysis techniques, and the BEI of each item was assessed to quantify their relative importance. This network provides insights into the relationship between family functioning and self-transcendence in breast cancer patients and potential targets for intervention.

### The relationship between different items of family functioning and self-transcendence

The cross-community edges in the network provides us an intuitive display of the underlying interaction pathways between family functioning and self-transcendence (36). Therefore, the crucial edges connecting the two communities are discussed to deepen our understanding of the fine-grained relationship between family functioning and self-transcendence. In the current network, each dimension of family functioning was related to some items of self-transcendence, and the nonzero correlation was mainly positive. F1 “Adaptation” and T1 “Enjoyment of hobbies” had the strongest correlation, which is in line with the results of a qualitative study (45): adequate family resources can increase a family's adaptability and an individual's ability to cope with a crisis and thereby maintain one's love for life. The reason for this may be that adequate material support through this process can provide patients with more opportunities for treatment and better care so that they have greater hope for their own life prospects (46). Conversely, when family resources are insufficient, such as when there is an economic burden, not only will patients experience anxiety but the emotional burden of family members will also increase (47). F2 “Participation” and T14 “Life enjoyment” had the strongest correlation, and F2-T14 was the strongest bridge connection among the cross-community correlations. This may be because, with the diagnosis of breast cancer, breast cancer patients will face a role reversal (48). Family communication has a very positive effect on patients' psycho-social adaptation (49), and family participation in treatment and life decision-making can also increase patients' treatment compliance and accelerate their role and life adaptation (50). On the other hand, a lack of communication between patients and family members was found to present an increased risk of poor adjustment (51). As breast cancer patients adapt to their roles, they also face the challenge of accepting physical changes as treatment progresses. In this study, we found the strongest correlation between F3 “Growth” and T5 “Acceptance of bodily changes”. Family growth refers to the acceptance and support of family members when patients wish to engage in new activities. Cancer patients mainly rely on family support during treatment, and good family support can help patients accept physical changes and reduce image anxiety (52, 53). Theoretical models have determined that social support from family may function as an antecedent of personal growth by facilitating acceptance of disability (54). Previous studies have found that cancer itself can make patients more emotionally dependent on their family members (55). The ability of family members to express emotions can help cancer patients reduce pain, increase family closeness (56), help reduce negative emotions, promote disease management and create an active lifestyle (57). This study found that F4 “Affection” and T14 “Life enjoyment” were most closely related, again providing support for the above study. In addition, the strongest correlation was found between F5 “Resolution” and T13 “Help acceptance”. This may be because family members are often a source of emotional support for the patient and because the presence of family members is a source of comfort to the patient, increasing his or her desire to live, adherence to treatment, and willingness to receive help from society (58).

In addition, we found some edges with strong links, which exist within each community, rather than between the communities. The edges with the strongest partial regularization correlation in the community of family functioning were between F4 “Affection” and F5 “Resolution”. The strong partial correlation between two nodes indicated that these two nodes had high co-occurrence. The reason may be that Chinese breast cancer patients in the treatment process mainly rely on family support and care (28). With the full understanding and support of their spouses, patients will feel spiritual satisfaction and pleasure, intimacy between couples will be increased, and the quality of marriage will be improved (59). Hofmann et al. (60) and Ahn and Kim (61) also found that in families with high levels of intimacy, the emotional bonds between family members are strong. Considering all of the above, it is understandable that there was a strong association between F4 “Affection” and F5 “Resolution” in family function.

In the self-transcendence community, the strongest partial correlation was between T4 “Adjustment to life” and T3 “Involvement with people”. Surgery, radiotherapy and chemotherapy are important methods for the treatment of breast cancer (62) and can cause image problems such as those due to breast loss (mastectomy), surgical chest scars, and hair loss, often leading to low self-esteem, social anxiety and social avoidance behavior that seriously affect the quality of life of women (63–65). Severe social anxiety and severe social avoidance behavior can lead to intense painful emotions and avoidance behaviors, which significantly weaken the individual's participation in the desired activities (66). This not only disrupts their lifestyle and daily routines but also makes it harder for them to adjust to their social life. Therefore, this association between T3 and T4 might be a point of intervention in the realization of self-transcendence.

### The intervention target of family functioning and self-transcendence

Bridges in the network plays an crucial role in the activation and maintenance of the connections between family functioning and self-transcendent, thus interventions targeting bridge nodes are most effective in strengthening the link between family functioning and self-transcendent (36, 67). The BEI index evaluated the role of a given node in the connection of family functioning and self transmitting, and the nodes with the highest BEIs were identified as bridges.

In the current network, F1 “Adaptation” had the largest BEI in the family functioning community. This is similar to Freud's (68) finding that family adaptation and affection are central to family functioning, except that the central role of adaptation is clearer through network analysis. This suggests that F1 “Adaptation” not only has the greatest impact on improving family functioning in patients with breast cancer but also has stronger associations with items of self-transcendence than other components. Thus, from a network perspective, targeting component F1, “Adaptation”, may be more effective for improving self-transcendence than targeting other components of family functioning. It is worth mentioning that this represents a hypothesis that should be tested in an experimental and clinical manner (69). From a conceptual perspective, family adaptation is an ability for families to engage in direct responses to the extensive demands of a stressor to restore factional stability and improve family satisfaction and wellbeing (70). Therefore, medical staff can guide patients to actively seek help from family members when they encounter problems and to talk about their feelings to family members to relieve psychological stress. At the same time, medical staff can also fully mobilize the enthusiasm of family members; remind family members to care for, accompany and care for patients; detect patients' physical and psychological problems in a timely manner; and take appropriate measures to solve them to improve the family's “adaptability” and improve family functions.

In the community of self-transcendence, the BEI of T14 “Life enjoyment” was the largest, indicating that this item had the most extensive and closest associations with other items in the community of family functioning. This item is more likely activated by family functioning so that it propagates activation throughout the self-transcendent network by connecting the edges of other items (60). It also provides an important potential target (71) for breast cancer patients to improve their level of self-transcendence. However, previous studies on the self-transcendence of breast cancer patients evaluated self-transcendence directly based on the total score, ignoring the effect of each item. Therefore, measures have been taken to help breast cancer patients adjust to the current pace of life to help them accept the disease in a positive way, to accept themselves, to find meaning in their existence and to achieve self-transcendence, which may be more effective than interventions against other items. In fact, from the moment the disease is diagnosed, the normal life trajectory of breast cancer patients may be disrupted. Studies (72, 73) have shown that 20% ~ 50% of breast cancer patients suffer from psychological distress during the whole period from diagnosis to treatment, which seriously affects their quality of life. Therefore, medical staff should provide psychological support to patients with breast cancer from the time of diagnosis to the whole period of treatment. Patients can be familiarized with the relevant knowledge of the disease through activities such as dissemination of scientific knowledge about the disease, interpretation of the treatment process by authoritative experts, emphasis of patients' psychological changes over time, provision of positive guidance, and help for patients in increasing their adaptability to painful events, thus improving their level of self-transcendence.

### Implications for clinical application

This study explored the relationship between family functioning and self-transcendence from the perspective of network and obtained fine-grained results. These results may shed light on future research aiming to develop theoretical understanding and provide several implications for interventions to meet the needs of mental health in patients with breast cancer, such as targeting F1 “Adaptation” and T14 “Life enjoyment” may be more effective at improving their level of self-transcendence than targeting other components of the network.

## Limitations

There are some limitations to our study. First, because cross-sectional data were used in this study to perform the analyses, we cannot ascertain the directionality of the edges. For instance, we cannot clarify whether the most central node triggers other nodes, is triggered by other nodes, or both. Thus, the causal relationship between nodes could not be determined. Time series data can be used to explore the temporal causality between nodes in future studies. Second, the network in this study estimated between-subject effects on a group level. Thus, it is possible that characteristics, such as centrality and network structure, may not remain the same on an individual level. In addition, different scales of family functioning may have different characteristics of the network structure, which can be further explored in future studies.

## Conclusion

In summary, our study provides an illustration of the richness and complexity of the associations involved in the structure of family functioning and self-transcendence in patients with breast cancer. In the network, different aspects of family functioning were positively correlated with different aspects of self-transcendence. From the perspective of network analysis, the nodes and the interrelations revealed in this study may support the clinical improvement of and interventions for self-transcendence in patients with breast cancer.

## Data availability statement

The original contributions presented in the study are included in the article/Supplementary material, further inquiries can be directed to the corresponding authors.

## Ethics statement

Written informed consent was obtained from the individual(s) for the publication of any potentially identifiable images or data included in this article.

## Author contributions

CH, TY, and YH proposed the experiment, designed the procedure, and did the most work of the manuscript. SW, BC, and XL gave useful comments to the experiment and helped to revise the manuscript for several times. CW, YL, SG, and LG helped to find participants and did the experiment work. All authors contributed to the article and approved the submitted version.

## Funding

This study was supported with grants from the Key Research and Development Plan of Shaanxi Province: General Projects - social development field (Grant Number: 2022SF-371).

## Conflict of interest

The authors declare that the research was conducted in the absence of any commercial or financial relationships that could be construed as a potential conflict of interest.

## Publisher's note

All claims expressed in this article are solely those of the authors and do not necessarily represent those of their affiliated organizations, or those of the publisher, the editors and the reviewers. Any product that may be evaluated in this article, or claim that may be made by its manufacturer, is not guaranteed or endorsed by the publisher.

## Abbreviations

Enjoyment of hobbies: In my present life, I enjoy my existing hobbies or interests
Acceptance of aging: As I grow older, I can still accept myself
Involvement with people: I still get involved with people or communities
Adjustment to life: I can make a good adjustment to my present life
Acceptance of bodily changes: I can accept and adjust to changes in my bodily functions
Experience sharing: I am willing to share my wisdom or experience with others
Meaning of the past: I can find meaning in my past experience
Helping others: I will help others in some ways
Interest in learning: My interest in learning never stops
Problem solving: When faced with something important, I can think beyond the problem and solve it
Death acceptance: I can accept that death is a part of life
Meaning in faith: I can find meaning in my faith
Help acceptance: When I am in need, I am willing to accept help from others
Life enjoyment: I enjoy the pace of my life right now
Letting go of the past: I can let go of the gains and losses of the past.
