# Supplementary material for: Relationship between family functioning and self-transcendence in patients with breast cancer: A network analysis
Source: Front Public Health. 2022 Nov 17;10:1028860. doi: 10.3389/fpubh.2022.1028860 (PMC9714448; doi:10.3389/fpubh.2022.1028860)
Supplement: Supplementary file 1 [file Table_1.docx]

Table S1 The [correlation](javascript:;) [matrix](javascript:;) in the network of family functioning and self-transcendence

|  | F1 | F2 | F3 | F4 | F5 | T1 | T2 | T3 | T4 | T5 | T6 | T7 | T8 | T9 | T10 | T11 | T12 | T13 | T14 | T15 |
| --- | --- | --- | --- | --- | --- | --- | --- | --- | --- | --- | --- | --- | --- | --- | --- | --- | --- | --- | --- | --- |
| F1 | 0.00 | 0.26 | 0.11 | 0.13 | 0.07 | 0.10 | 0.00 | 0.03 | 0.01 | 0.00 | 0.01 | 0.00 | 0.00 | 0.00 | 0.00 | 0.06 | 0.00 | 0.09 | 0.00 | 0.00 |
| F2 | 0.26 | 0.00 | 0.09 | 0.16 | 0.24 | 0.00 | 0.00 | 0.00 | 0.00 | 0.00 | 0.00 | 0.00 | 0.00 | 0.00 | 0.00 | 0.00 | 0.00 | 0.00 | 0.14 | 0.00 |
| F3 | 0.11 | 0.09 | 0.00 | 0.24 | 0.05 | 0.00 | 0.00 | 0.00 | 0.00 | 0.10 | 0.01 | 0.05 | 0.00 | 0.00 | 0.00 | 0.00 | 0.00 | 0.00 | 0.02 | 0.00 |
| F4 | 0.13 | 0.16 | 0.24 | 0.00 | 0.29 | 0.00 | 0.00 | 0.00 | 0.00 | 0.02 | 0.00 | 0.00 | 0.00 | 0.00 | 0.00 | 0.05 | 0.03 | 0.00 | 0.10 | 0.00 |
| F5 | 0.07 | 0.24 | 0.05 | 0.29 | 0.00 | 0.02 | 0.00 | 0.00 | 0.00 | 0.00 | 0.00 | 0.00 | 0.00 | 0.00 | 0.00 | 0.00 | 0.00 | 0.03 | 0.01 | 0.00 |
| T1 | 0.10 | 0.00 | 0.00 | 0.00 | 0.02 | 0.00 | 0.25 | 0.25 | 0.03 | 0.10 | 0.00 | 0.03 | 0.08 | 0.00 | 0.00 | 0.00 | 0.01 | 0.01 | 0.00 | 0.00 |
| T2 | 0.00 | 0.00 | 0.00 | 0.00 | 0.00 | 0.25 | 0.00 | 0.04 | 0.17 | 0.05 | 0.00 | 0.00 | 0.02 | 0.00 | 0.00 | 0.07 | 0.00 | 0.00 | 0.06 | 0.07 |
| T3 | 0.03 | 0.00 | 0.00 | 0.00 | 0.00 | 0.25 | 0.04 | 0.00 | 0.33 | 0.01 | 0.09 | 0.05 | 0.09 | 0.04 | 0.00 | 0.00 | 0.00 | 0.00 | 0.07 | 0.00 |
| T4 | 0.01 | 0.00 | 0.00 | 0.00 | 0.00 | 0.03 | 0.17 | 0.33 | 0.00 | 0.27 | 0.00 | 0.11 | 0.00 | 0.01 | 0.00 | 0.04 | 0.08 | 0.00 | 0.17 | 0.06 |
| T5 | 0.00 | 0.00 | 0.10 | 0.02 | 0.00 | 0.10 | 0.05 | 0.01 | 0.27 | 0.00 | 0.12 | 0.00 | 0.00 | 0.00 | 0.12 | 0.05 | 0.00 | 0.00 | 0.00 | 0.15 |
| T6 | 0.01 | 0.00 | 0.01 | 0.00 | 0.00 | 0.00 | 0.00 | 0.09 | 0.00 | 0.12 | 0.00 | 0.20 | 0.21 | 0.00 | 0.00 | 0.00 | 0.00 | 0.03 | 0.00 | 0.06 |
| T7 | 0.00 | 0.00 | 0.05 | 0.00 | 0.00 | 0.03 | 0.00 | 0.05 | 0.11 | 0.00 | 0.20 | 0.00 | 0.08 | 0.20 | 0.18 | 0.00 | 0.05 | 0.01 | 0.05 | 0.01 |
| T8 | 0.00 | 0.00 | 0.00 | 0.00 | 0.00 | 0.08 | 0.02 | 0.09 | 0.00 | 0.00 | 0.21 | 0.08 | 0.00 | 0.06 | 0.07 | 0.00 | 0.05 | 0.10 | 0.00 | 0.00 |
| T9 | 0.00 | 0.00 | 0.00 | 0.00 | 0.00 | 0.00 | 0.00 | 0.04 | 0.01 | 0.00 | 0.00 | 0.20 | 0.06 | 0.00 | 0.17 | 0.06 | 0.07 | 0.00 | 0.14 | 0.00 |
| T10 | 0.00 | 0.00 | 0.00 | 0.00 | 0.00 | 0.00 | 0.00 | 0.00 | 0.00 | 0.12 | 0.00 | 0.18 | 0.07 | 0.17 | 0.00 | 0.09 | 0.24 | 0.00 | 0.00 | 0.12 |
| T11 | 0.06 | 0.00 | 0.00 | 0.05 | 0.00 | 0.00 | 0.07 | 0.00 | 0.04 | 0.05 | 0.00 | 0.00 | 0.00 | 0.06 | 0.09 | 0.00 | 0.15 | 0.03 | 0.00 | 0.05 |
| T12 | 0.00 | 0.00 | 0.00 | 0.03 | 0.00 | 0.01 | 0.00 | 0.00 | 0.08 | 0.00 | 0.00 | 0.05 | 0.05 | 0.07 | 0.24 | 0.15 | 0.00 | 0.00 | 0.07 | 0.13 |
| T13 | 0.09 | 0.00 | 0.00 | 0.00 | 0.03 | 0.01 | 0.00 | 0.00 | 0.00 | 0.00 | 0.03 | 0.01 | 0.10 | 0.00 | 0.00 | 0.03 | 0.00 | 0.00 | 0.00 | 0.06 |
| T14 | 0.00 | 0.14 | 0.02 | 0.10 | 0.01 | 0.00 | 0.06 | 0.07 | 0.17 | 0.00 | 0.00 | 0.05 | 0.00 | 0.14 | 0.00 | 0.00 | 0.07 | 0.00 | 0.00 | 0.04 |
| T15 | 0.00 | 0.00 | 0.00 | 0.00 | 0.00 | 0.00 | 0.07 | 0.00 | 0.06 | 0.15 | 0.06 | 0.01 | 0.00 | 0.00 | 0.12 | 0.05 | 0.13 | 0.06 | 0.04 | 0.00 |
