# Supplementary figures and images for: Relationship between family functioning and self-transcendence in patients with breast cancer: A network analysis
Source: Front Public Health. 2022 Nov 17;10:1028860. doi: 10.3389/fpubh.2022.1028860 (PMC9714448; doi:10.3389/fpubh.2022.1028860)

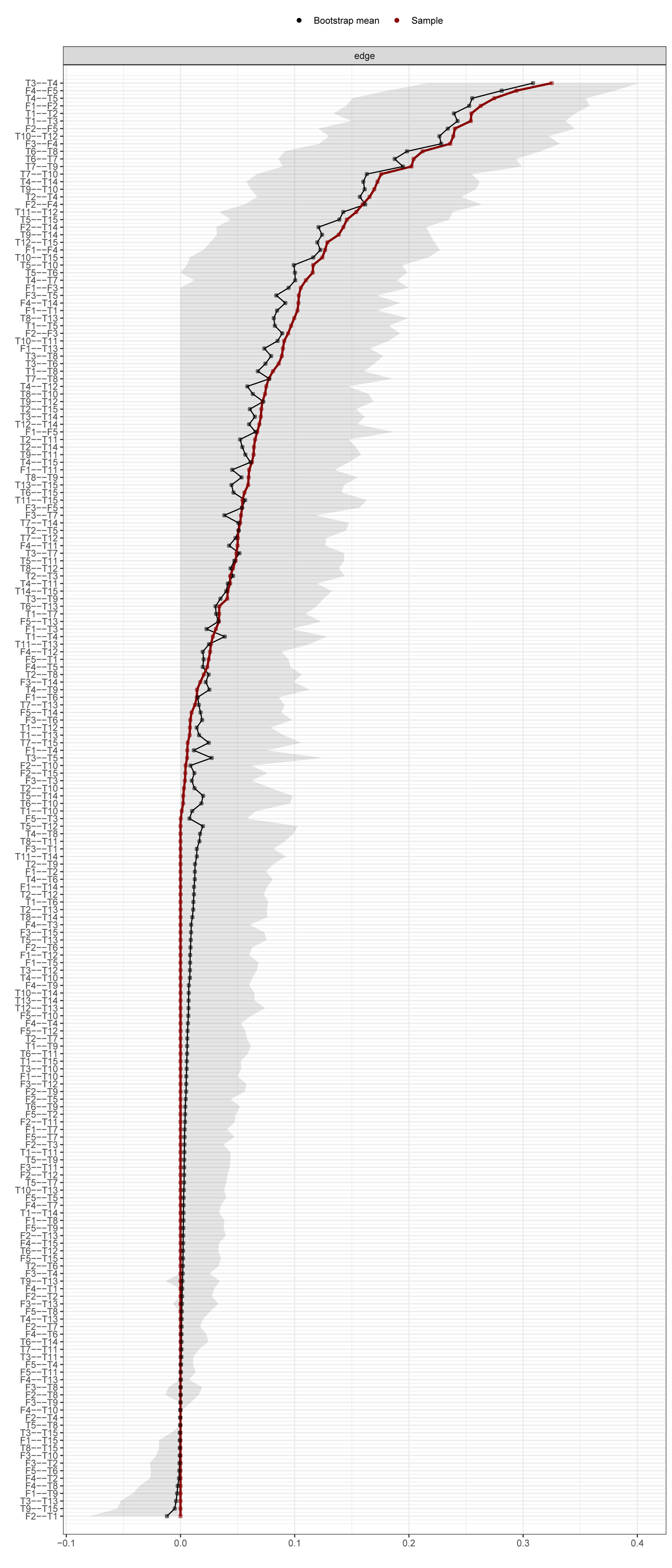

Supplement: Supplementary file 2 [file Image_1.JPEG]

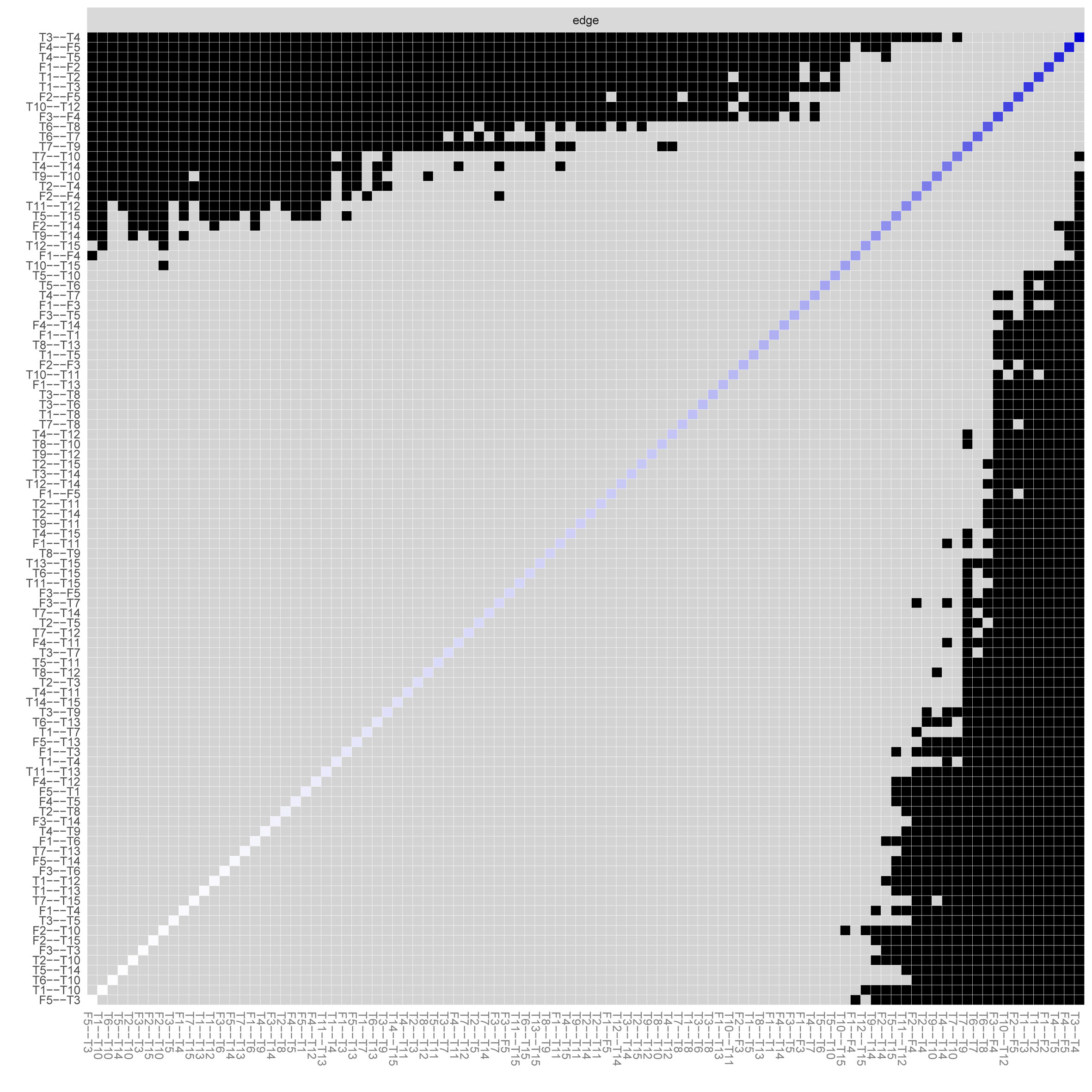

Supplement: Supplementary file 3 [file Image_2.JPEG]

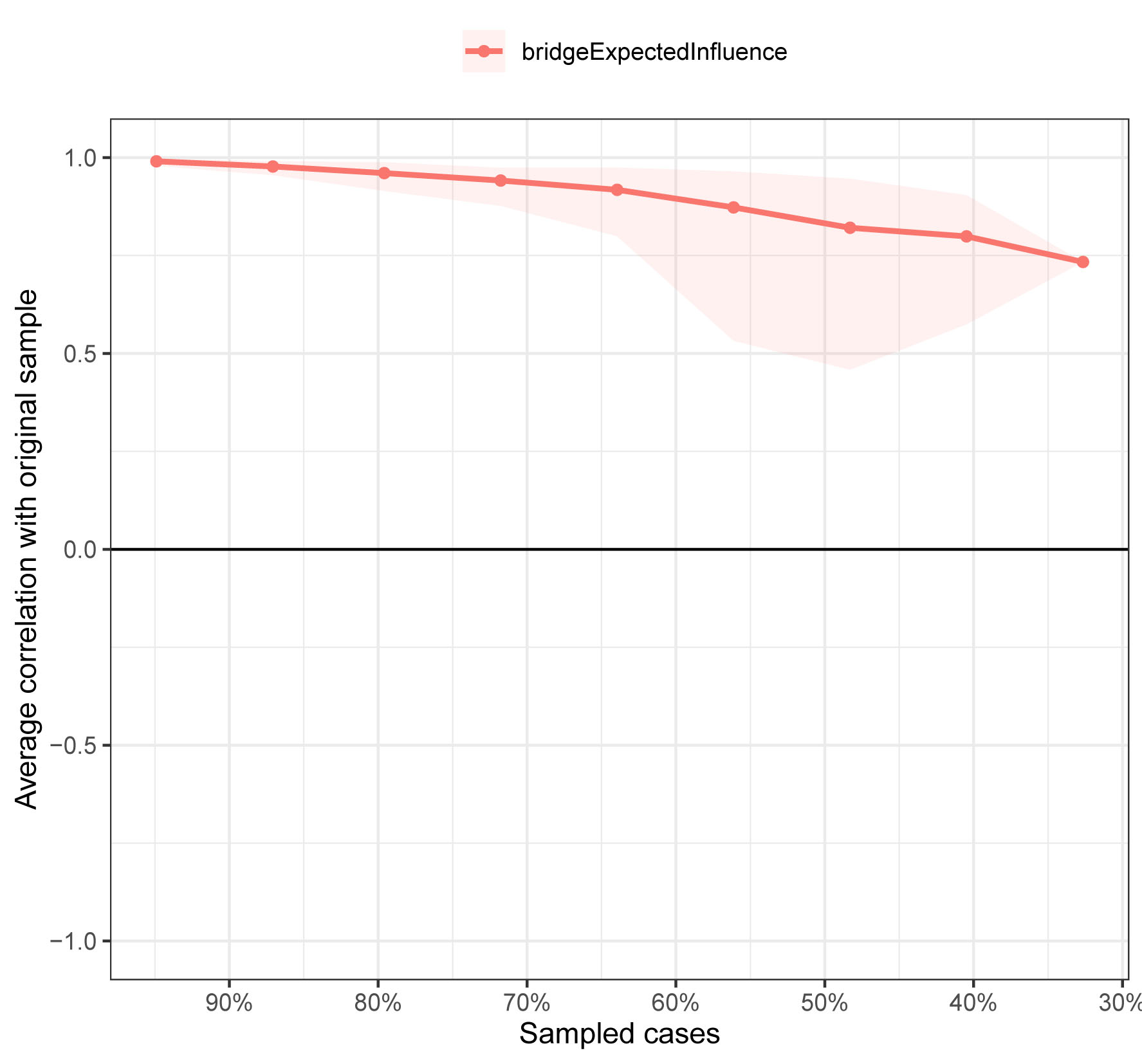

Supplement: Supplementary file 4 [file Image_3.JPEG]

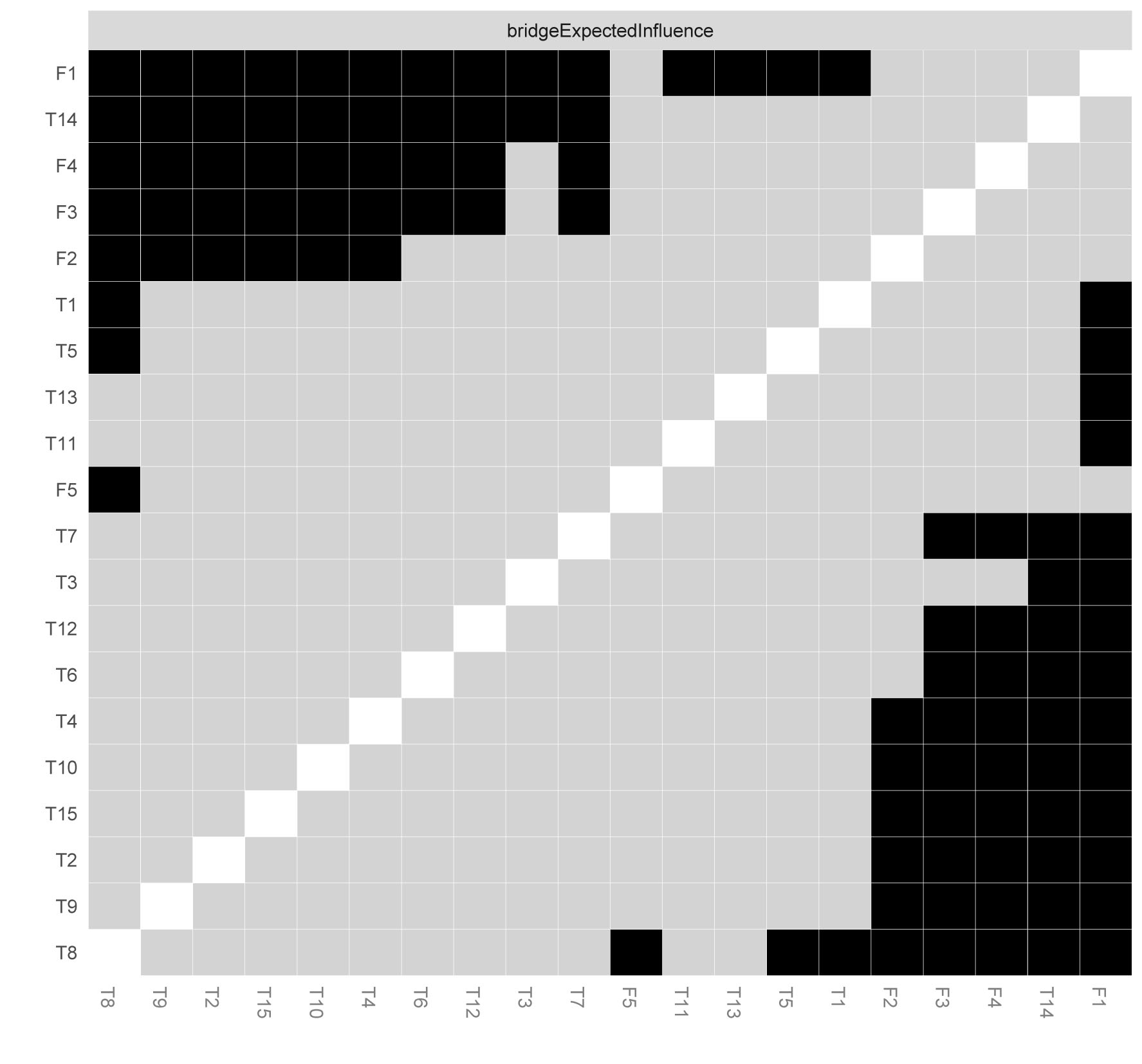

Supplement: Supplementary file 5 [file Image_4.JPEG]
